# A 10 year follow-up study after Roux-Elmslie-Trillat treatment for cases of patellar instability

**DOI:** 10.1186/1471-2474-12-48

**Published:** 2011-02-18

**Authors:** Stefan Endres, Axel Wilke

**Affiliations:** 1 Department of Orthopaedic Surgery Elisabeth-Klinik Bigge/Olsberg, Heinrich-Sommer-Strasse 4, 59939 Olsberg, Germany

## Abstract

**Background:**

A retrospective study concerning patients presenting with patella instability, treated using a Roux-Elmslie-Trillat reconstruction operation and followed up for 10 years following surgery, is presented.

**Methods:**

Pre-operative and follow-up radiographic evaluation included the weight-bearing anteroposterior and merchant views. Evaluation was carried out using the Insall-Salvati index, sulcus and congruence angle. The Roux-Elmslie-Trillat reconstruction operation was performed on 18 patients. The clinical evaluation at follow-up was performed using the Knee-Society-Score (KSS) and Tegner-Score.

**Results:**

Subjective results of the operation were classed as excellent or good in 16 of the 18 patients ten years after surgery; persistent instability of the patella was recorded in only one of the 18 patients. The majority of patients returned to the same level of sporting activity after surgery as they had participated in before injury.

**Conclusions:**

The Roux-Elmslie-Trillat procedure could be recommended in cases presenting with an increased q-angle, trochlea dysplasia or failed soft tissue surgery. In the present study the majority of patients report a return to previous sporting activity ten years after surgery.

## Background

Patellofemoral instability is generally defined as acute or chronic. Acute instability refers to a primary, traumatic episode in which the patella dislocates laterally, while chronic instability denotes recurrent dislocations. Medial dislocations are rare and are typically iatrogenic.

According to the Dejour classification, patients presenting with patellofemoral instability can demonstrate several abnormalities in the patellofemoral joint including hypoplastic patella, genu recurvatum, trochlear dysplasia, dysplastic lateral femur condyle, patella alta, ligament laxity and excessive quadricep angles [1].

Several interventions to correct the underlying pathology have been described in the literature [2,3]. However, in most cases, a single procedure does not result in a satisfactory outcome owing to the complexity of the disorder. Clinical results vary in terms of patient satisfaction from 20 to 70% [4], depending on the operating procedure used.

Therefore, the long-term results (10 year follow-up) of surgery were assessed with the main focus on the clinical outcome after a Roux-Elmslie-Trillat procedure in cases of patellar instability.

## Methods

In a retrospective study (approved by the Ethics Committee of the University of Marburg, Germany) the results of a Roux-Elmslie-Trillat procedure were examined after a mean follow up of 9.7 years (Range 9.1-11.6 years; SD: 1.8).

Between 1998 and 2003 the Roux-Elmslie-Trillat procedure was carried out on 23 knees (23 patients) with recurrent or habitual dislocation of the patella and lateral hypercompression syndrome of the patella with subluxation. Conservative treatment for a minimum of three months (re-education of the quadriceps) had proved unsuccessful in these patients. Eighteen knees from 18 patients were assessed before surgical intervention and the clinical outcome was assessed after surgical intervention.

Formal exclusion criteria were traumatic initial luxations, open proximal tibial epiphyseal gaps (risk of genu recurvatum) and clearly defined genu valgum (> 10°).

Pre-operative findings were obtained from a physical examination and conventional radiological diagnostics. Reports concerning findings from post-operatively conducted three-monthly checks and operations were compiled.

Patents were excluded if documentation was incomplete, if further operations had been conducted or if there were traumata (e.g. meniscus lesion, patellar tendon rupture, fractures) on the operated knee joint.

The results of the follow-up examination were evaluated via a physical examination, a patient interview based on standardised knee scores and conventional radiological diagnostics.

The main focus of this retrospective study was the evaluation of the clinical outcome; the radiological evaluation was carried out for secondary outcome measurements.

The patients were clinically evaluated using Tegner and Knee-Society scores (KSS), a clinical function-oriented questionnaire that evaluates data gathered in relation to the knee joint itself and the patient's capabilities in terms of routine activities such as climbing stairs and exercise or engaging in competitive sport.

The clinical section of the KSS involves quantifying parameters of pain, function and stability, while considering other clinical entities such as the level of exercise and mechanical axis deviation as defined by a varus or turned outward position. In the functional section of the KSS, the patient's load capacity on the knee joint is evaluated; scores of 80 and above are considered to be excellent, 79 to 70 considered good, 69 to 60 classed as fair and less than 60 is considered poor.

**Figure 1:**
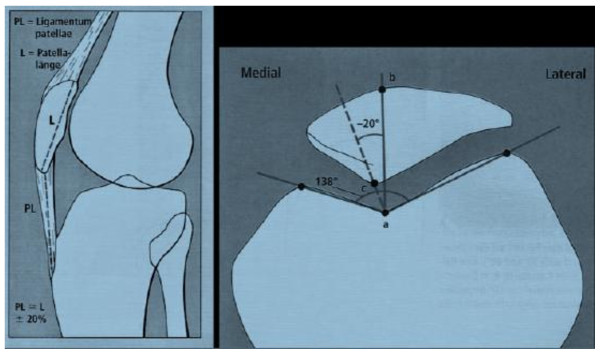
**Example of the determination the Insall-Salvati index, sulcus and congruence angle**.

Radiographs taken before and after surgery in anteroposterior (AP), lateral and "merchant view" projections were used to determine the Insall-Salvati index of patellar height, the congruence and the sulcus angle, being indicative of the shape of the patella and the femoral groove, as an index of subluxation (Figure 1).

Data were collated and interpreted by the author to minimise intra-observer error.

Eighteen patients with patellar instability [relapsed traumatic or habitual patellar luxation (14/18) and patellar subluxation in combination with patellar lateralisation (4/18)] underwent surgery.

The median age of patients was 28.2 years (17-48; SD: 12.7), with 14 female patients and four males. All patients engaged in regular physical activities including jogging, hiking, walking, swimming, skiing and tennis, and other types of exercise in individual cases. The maximum body mass index (BMI) was 29.4, with an average of 24.5 (SD: 4.7).

### Operational procedure

All operations were carried out by the senior author of this manuscript (AW). The operation took an average of 72 minutes (range: 38-90 minutes; SD: 16.91). Each of the patients was given a 1.5g perioperative single shot of the prophylactic antibiotic Cefuroxim. Pre-operative MRI demonstrated no pathology of the knee joint. Arthroscopy, carried out before the Roux-Elmslie-Trillat procedure, confirmed that other than patellar instability there was normal anatomy of the knee joint.

An anterolateral approach was chosen as the access point for the Roux-Elmslie-Trillat procedure. The tuberositas tibiae was detached at three points (medial, lateral, proximal), resulting in a distally bony bridge being left intact. After medialising the tuberositas tibiae of 10-20 mm (depending on the position of the patella) it was fixed in position with two 6.5mm cancellous screws and a washer.

In addition, a release of the lateral retinaculum and a medial capsular plication was carried out. After inserting a Redon drainage system, the wound was sealed in layers, and a sterile compression dressing applied.

### Operative findings

At the time of surgery eight knees had grade-I changes, six knees had grade-II changes, three knees had grade-III changes and one had an osteochondral flake fracture which required to be removed. No knees had severe degenerative changes at time of the surgery.

### Post-treatment

Post-operatively, the patients were mobilised with crutches from one day following the operation. Isometric exercises of the femoral muscles were conducted on the first day following the operation. For exercise training and thrombosis prevention, a CPM (continuous passive motion) with flexion limitation of 60° was used. The Redon drainage tube was removed two days after the operation. The suture material was removed ten days after the operation. At the beginning of the sixth postoperative week there was a rapid increase in load and limited flexion of 60° after a radiological check, demonstrating healing of the medialised bone block.

### Statistical analysis

The data were analysed by a Wilcoxon-Whitney-Mann test for unconnected samples, as the requirements for a t-test (α1 = σ2) were not fulfilled. The pre- and post-operative data were compared to investigate whether there was (H1: μ1 > μ2 or μ1 < μ2) or no difference (H0: μ1 = μ2). P-values ≤ 0.05 were regarded as statistically significant. All readings were provided as average values together with the appropriate standard deviation. The post-test results were related to the pre-operative knee evaluation sheet point readings.

## Results

The results presented refer exclusively to subsequently examined patients of whom eleven were operated on the right knee and seven on the left.

### Complications

Post-operative swelling of the joint occurred in five patients (5/18) but intervention was not required and none of the patients presented with a break in a bone splint or tear in the tuberositas tibiae. No pseudoarthrosis formation of the osteotomy was observed or any osteosynthesis failure. Furthermore, there was no evidence of infection, deep leg vein thrombosis, embolism or secondary bleeding that required surgical treatment.

One patient continued to present with an unstable patella with a tendency to subluxation, but this was observed after 38 months. In this case, a further operation was carried out with an arthroscopy to identify the intra-articular accompanying injuries, and medial pinning of the patella with a semitendinosus transplant was carried out [5].

### Score results - KSS and Tegner Score (Table 1)

**Table 1:** Functional Score after a mean post-operative follow up of 9.7 years

Functional Scoring System	Mean preoperative Score (95% CI)	Mean postoperative Score (95% CI)	Statistical significance
Knee Score	74.5	87.7	0.01
Function Score	82	88.8	0.01
Tegner Score	3.4	4.6	0.05

The majority of patients demonstrated definite post-operative improvement in function, activity and mobility of the operated knee joint. Subjective results of the operation were excellent or good in 16 of the 18 patients ten years after surgery. Most patients were able to return to the same level of sporting activity that they engaged in before injury.

The average knee score (KSS) improved significantly (p < 0.01) during the ten year follow-up: 74.5 pre-operatively (range: 50-95; SD: 13.6) to 87.7 ten years after the operation (range: 68-100; SD: 9.2). One patient reported deterioration, which was due to persistent instability of the patella with subsequent OP.

The average function score (KSS) improved significantly (p < 0.01) from 82 pre-operatively (range: 30-100; SD: 19.6) to 88.8 ten years after the operation (range: 70-100; SD: 12.3). Furthermore, the average activity score (Tegner-Score) increased significantly (p < 0.05) from 3.4 pre-operatively to 4.6 ten years after the operation; an initial drop to 2.8 was evident three months after the operation.

Eleven of the 18 patients who presented with a poor score after three months blamed the prominence of the screw osteosynthesis in the area of the tuberositas tibiae. The focus concerned symptoms that were noticeable particularly when kneeling, but others associated with general activities were noted. One year after the Roux-Elmslie-Trillat procedure, the screws were removed. Those patients who reported tenderness and pain due to the screw osteosynthesis demonstrated an improvement in the KSS and Tegner activity scores after removal.

### Radiological analysis (Table 2)

**Table 2:** Radiographic analyses after a mean postoperative follow up of 9.7 years

Radiographic anaylses	Mean preoperative Value (95% CI)	Mean postoperative Value (95% CI)	Statistical significance
Insall-Salvati Index	0.85	0.89	> 0.05
Congruence angle	+ 7°	+ 0.8°	0.01
Sulcus angle	143°	143.31°	> 0.05

Radiological evaluation demonstrated a definite correction of the congruence angle. The average pre-operative congruence angle measured + 7° (range: 2° to + 36° SD: 7.5°); the average post-operative congruence angle was significantly reduced (p < 0.01) to + 0.8° (range: - 7° to + 6° SD: 3.76°).

The sulcus angle demonstrated no significant changes because no corrective measures were carried out on the trochlea femoris (average preoperative reading: 143° SD: 6.32° vs. average postoperative reading: 143.31° SD: 5.95°).

As a result of the operation, the height of the patella and the average Insall-Salvati index did change significantly: preoperative 0.85 (range: 0.72-1.07; SD: 0.06); postoperative 0.89 (range: 0.7-1.07; SD: 0.08). No progressive arthritic changes were observed during the course of the follow-up.

## Discussion

The term patellofemoral instability encompasses several abnormal knee joint disorders, ranging from acute patellar luxation to chronic instability of the patella, to patellar decentration in the patellofemoral groove. Various operative methods [2,3,6] have been used in an attempt to correct these factors. In the literature, several conservative and operative treatment options have been described [7].

The majority of operative techniques can be classified as lateral release, restoration of proximal alignment, restoration of distal alignment and a combination of these procedures. The principle of tuberositas tibiae transfer to treat habitual patellar luxation or patellofemoral pain is attributed to Elmslie. It was subsequently popularised by Trillat [8] and its original operational model has been repeatedly modified.

One of the best known modifications is the Roux-Elmslie-Trillat technique. This technique involves releasing the lateral retinaculum, a medial capsular plication and medialisation of the tuberositas tibiae [9].

Such distal bony procedures have an established success rate and provide an appropriate alternative for patients with closed growth plates and an abnormal lateral position of the tibial tuberosity, have been subjected to previous failed soft tissue procedures, have documented patellofemoral arthritis, or present with generalized hyperlaxity. Each of these conditions is likely to result in the failure of a purely proximal soft tissue procedure.

There are few published data concerning the initial evaluation of results. Kumar et al. reported no tendency to reluxation in nine patients (100%) in an average follow-up examination period of three years [10].

Published data concerning long-term follow-up examinations are rare. In a Medline search going back 35 years, the maximum average follow-up examination period that had been published was 13 years [11]. The study concerned 14 patients, and 15 Roux-Elmslie-Trillat operations carried out on patients with acute patellar luxation or subluxation were evaluated. It should be noted that this study included patients that were evaluated previously in a study by Cox et al. [9].

Current knowledge indicates only two studies that have evaluated a follow-up examination period beyond 10 years [11,12]. Nakagawa et al. reported a subluxation/reluxation rate of 13% (six of 45 operated knees) in an average follow-up examination period of 13.5 years, where 14 patients and 15 Roux-Elmslie-Trillat operations were carried out on patients with acute patellar luxation or subluxation [11]. This study also included patients that were previously evaluated in a study by Cox et al. [9].

Carney et al., whose average follow-up examination period was 26 years, demonstrated that the long-term results achieved were comparable with those after three years. Furthermore, the incidence of reluxation of the patella or subluxation was 7% (one out of 15 knees), much lower than the 13% of patients subjected to a follow-up examination by Nakagawa.

The incidence of reluxation in the present follow-up study, 1/18 (0.02%), was significantly lower than those reported by Carney et al. or Nakagawa. The scores presented in this study were recorded over an average follow-up examination period of 9.7 years. The average Tegner score improved significantly from 3.4 pre-operatively to 4.6 three years post-operation, the average function score from the KSS improved significantly from 82 pre-operatively to 88.8 (p < 0.01) and the knee score from the KSS improved significantly from 74.5 pre-operatively to 87.7 post-operatively (p < 0.01).

These scores provide a statistical outline of the operation results, and allow the functional results to be predicted. The radiological evaluation produced a significant correction (p < 0.01) of the congruence angle (+ 7° to + 0.8°), which has crucial influence on stability in the patellofemoral joint. A change in the position of the patella, in terms of a shift up or down, was avoided by this operational procedure. Therefore, no change in the Insall-Salvati index occurred. Radiological examination demonstrated no progressive arthritic changes in the patient collective. Furthermore, no patient required revision surgery due to progressive degenerative changes.

In contrast to Mirroneau [13] and Deburge and Chambat [14], who provided evidence of deterioration in terms of pain after conducting an Elmslie-Trillat operation, no patient in the present study reported post-operative worsening of pain, with the exception of the patient who required further surgery due to the reluxation event. All other patients reported an improvement in pain symptoms.

The results of the surgical procedure presented in this study are better than the medium to long-term clinical results reported in the literature following surgical treatment of patellofemoral instability via a single procedure [15], which vary between 20 and 70% in terms of patient satisfaction [4].

Grana and O'Donoghue [16] achieved 83% patient satisfaction with the Hauser technique but the rate of post-operative complications was recorded as 29% and the reluxation rate of the patella was 5%. Chrisman et al. [17] achieved 72% satisfactory results with the Hauser technique and 93% with the Roux-Goldthwait technique. Fielding et al. [18] and Trillat et al. [8] reported a shift to 73% and 78% satisfactory results, respectively, with operative interventions on tuberositas tibiae. Cox [9] reported 66% satisfactory long-term results after operating on patellar luxation using the Roux-Elmslie-Trillat technique.

However, critical observations of the study by Arnbjornsson et al. [19] and Marcacci et al. [20] should be noted. These studies demonstrated no significant clinical or radiological differences between patients treated operatively and conservatively during a long-term follow-up. However, in these trials a single procedure was used and the underlying pathology was not considered.

Different findings and clinical results have been published with regards to treating patellofemoral instability, probably because a multitude of factors can cause this condition. Operative correction of a single factor may not lead to the desired result. For example, Aglietti et al. reported a tendency to reluxation of up to 44% after lateral release alone [7]. Furthermore, Sherman et al. reported poor results for 25% of patients following arthroscopically conducted lateral release [21].

In contrast, the combination of lateral release and a medial capsular plication results in high patient satisfaction (95%), as demonstrated by Nam et al.. The incidence of reluxation in this study was reported as 4% with an average follow-up of 4.4 years [22]. These favourable results after combined soft tissue intervention were confirmed in studies by Scuderi et al. and Sargent et al. [23,24].

The operative correction of trochlear dysplasia, regarded as a major cause of patellofemoral instability, is common [25]. The very complex operative technique and initial results do not compare with the findings of this study.

A disadvantage of the majority of studies is that they comprise a comparatively small patient collective. Furthermore, the present research concerns a retrospective study, which was not compared with a control group. Comparisons with other follow-up studies are difficult, as various patient inclusion criteria and measuring instruments were used in each case. However, the present study demonstrates that using the Roux-Elsmlie-Trillat procedure for patellar instability can produce excellent long term results.

## Conclusions

It is imperative that individual pre-disposing factors are considered before surgery, and conservative treatment of patellar instability should be the first choice for treatment as in the majority of cases it produces good results [26].

In cases of patella instability due to an increased q-angle, trochlea dysplasia or after failed soft tissue surgery, where conservative treatment has been ineffective, the Roux-Elmslie-Trillat procedure produces good long term results. Furthermore, the procedure prevents recurring dislocation of the patella, which is decisive for the clinical success of the treatment.

## Competing interests

The authors declare that they have no competing interests.

### Financial competing interests

- In the past five years have you received reimbursements, fees, funding, or salary from an organization that may in any way gain or lose financially from the publication of this manuscript, either now or in the future? Is such an organization financing this manuscript (including the article-processing charge)? If so, please specify.

No.

- Do you hold any stocks or shares in an organization that may in any way gain or lose financially from the publication of this manuscript, either now or in the future? If so, please specify.

No.

- Do you hold or are you currently applying for any patents relating to the content of the manuscript? Have you received reimbursements, fees, funding, or salary from an organization that holds or has applied for patents relating to the content of the manuscript? If so, please specify.

No.

- Do you have any other financial competing interests? If so, please specify.

No.

### Non-financial competing interests

- Are there any non-financial competing interests (political, personal, religious, ideological, academic, intellectual, commercial or any other) to declare in relation to this manuscript? If so, please specify.

No.

## Authors' contributions

SE carried out the follow up study and drafted the manuscript. AW participated in the design of the study and in its coordination, and helped to draft the manuscript. All authors read and approved the final manuscript.

## Pre-publication history

The pre-publication history for this paper can be accessed here:

http://www.biomedcentral.com/1471-2474/12/48/prepub
